# Efficacy and safety of CD19 combined with CD22 or CD20 chimeric antigen receptor T-cell therapy for hematological malignancies

**DOI:** 10.3389/fimmu.2025.1577360

**Published:** 2025-05-13

**Authors:** Xiaoshuang Yuan, Feiqing Wang, Peng Zhao, Bo Yang, Xu Yang, Ting Tian, Bingbing Li, Guangyang Liu, Sanbin Wang, Dongxin Tang, Zhixu He, Yanju Li, Yang Liu

**Affiliations:** ^1^ Department of Hematology Oncology, Affiliated Hospital of Guizhou Medical University, Guiyang, Guizhou, China; ^2^ Clinical Medical Research Center, The First Affiliated Hospital of Guizhou University of Traditional Chinese Medicine, Guiyang, Guizhou, China; ^3^ Academy of Medical Engineering and Translational Medicine, Tianjin University, Tianjin, China; ^4^ Department of Hematology, The 920th Hospital of Joint Logistics Support Force, Kunming, Yunnan, China; ^5^ Center of Tissue Engineering and Stem Cell Research, Guizhou Medical University, Guiyang, Guizhou, China

**Keywords:** car-t, hematological malignancies, chimeric antigen receptor, immunotherapy, cancer therapy

## Abstract

**Background:**

CD19 combined with CD22 or CD20 therapy is a promising immunotherapy approach for the treatment of hematological malignancies. Dual-targeted CD19/CD22 CAR T and CD19/CD22 CAR T-cell therapy are currently being evaluated in clinical trials, and the extent of improvement using CD19 in combination with dual-targeted therapy has not yet been determined. To compare the differences between the two in the treatment of hematological tumors, this study summarized the available evidence. To evaluate and compare the efficacy and safety of CD19-combined CD22 and CD19-combined CD20 CAR T-cell therapy.

**Methods:**

Data from 13 clinical studies that included 628 patients with hematological malignancies were extracted and analyzed based on a set of inclusion and exclusion criteria. The primary efficacy outcomes were overall response rate (ORR), complete response (CR) rate, partial response (PR) rate, overall survival (OS) rate and minimal residual disease (MRD)-negative response rate. The safety outcomes were cytokine release syndrome (CRS) rate and immune effector cell-associated neurotoxicity syndrome (ICANS) rate.

**Results:**

For CD19 combined with CD22 CAR T-cell therapy, the ORR was 83.7%; CR, 78.0%; PR, 20.7%, OS, 78.7%; MRD-negative response rate, 82.3%; incidence of CRS, 58.2%; ICANS, 7.7%. For CD19 combined with CD20 CAR T-cell therapy, the ORR was 80.3%; CR, 68.2%; PR, 10.9%; OS, 76.8%; incidence of CRS, 54.5%; ICANS, 21%. Subgroup analysis indicated that the PR of CD19 combined with CD22 was significantly greater than that of CD19 combined with CD20, and the incidence of ICANS was significantly lower with the CD19+CD22 CAR-T combination.

**Conclusion:**

The data from this study suggest that CD19 combined with CD22 CAR T-cell therapy had a higher partial response rate in the treatment of hematologic malignancies and higher safety profile in the occurrence of ICANS than CD19 combined with CD20. These data provide an important clinical basis for the development of new therapeutic targets and the construction of therapeutic methods for the treatment of hematologic malignancies, and broaden our understanding of CD19 dual-targeted CAR T therapy.

## 1 Introduction

The treatment of hematological malignancies has traditionally primarily involved chemotherapy, radiotherapy, and hematopoietic stem cell transplantation. Advancements in tumor immunology have catalyzed the development of transformative immune-targeted therapies, including monoclonal antibodies, bispecific antibodies, antibody-drug conjugates (ADCs), and chimeric antigen receptor (CAR) T-cell therapy. These innovative modalities now represent a paradigm shift in oncology, offering precision-driven strategies to combat malignant tumors with enhanced therapeutic efficacy and reduced off-target effects (1). As a state-of-the-art approach, CAR T-cell therapy has achieved remarkable success in the treatment of refractory hematological malignancies (2). In CAR T-cell therapy, a patient’s T-cells are genetically engineered to express the chimeric antigen receptor (CAR) and then adoptively transferred back into the patient. Through genetic engineering, CAR T-cells are designed to leverage tumor-associated surface antigens as molecular targets. This enables precision-guided recognition and subsequent activation of cytotoxic mechanisms, effectively eliminating malignant cells via antigen-specific binding while minimizing off-tumor toxicity (3). Various target antigens of CAR T-cells have been reported in the literature, as discussed below.

CD19 is an apt target antigen for CAR T-cell immunotherapy in B-cell malignancies because it is expressed from early B-cell precursors (such as lymphoblastocytes) to mature B-cells (4–6). CAR T-cell therapy targeting CD19 has shown significant clinical efficacy in the treatment of relapsed or refractory B-cell malignancies (7). For example, in a study involving 3,421 patients with hematologic malignancies, the overall response rate reached 75% (8). However, despite achieving CR, nearly half of patients relapse within the first year post-treatment. One of the primary reasons for this relapse or treatment failure is the mutation or loss of CD19 (9). To overcome this limitation, researchers have explored alternative CAR T-cell therapies targeting novel antigens across diverse indications, with preliminary trials demonstrating encouraging clinical outcomes. Notably, CD20 and CD22—frequently co-expressed with CD19 in B-cell malignancies—have emerged as viable alternatives for CAR T-cell therapy, particularly in cases of CD19 antigen escape. CD22 is expressed in both normal and malignant B-cells, and this makes it a potential alternative to CD19 (10, 11). Moreover, CD22 has been found in the protocells of more than 90% of B-cell acute lymphoblastic leukemia/lymphoma cases (12, 13). Qin et al. developed a range of loop CD19/CD22 CAR-T constructs (LoopCAR6). They conducted *in vivo* experiments with LoopCAR6, achieving optimal transduction efficiency and cytokine production. Their findings revealed that LoopCAR6 exhibits a tumor-clearing effect on patient-derived xenografts exhibiting resistance to CD19 CAR T-cell therapy (14). CD20 is a surface protein that is commonly expressed in B-cells and rarely occurs in other tissues. This makes it an ideal target for immunotherapy against B-cell-derived malignancies. CD20 and CD22 CAR-T immunotherapies have achieved good results in hematological malignancies, including cases in which patients have previously received CD19 CAR T-cell therapy (15).

Evidence from preclinical models of solid tumors indicates that dual CAR T-cells may demonstrate synergistic effects, thereby enhancing response rates compared to those achieved by targeting a single antigen (16, 17). In 2016, Zah and collaborators pioneeringly introduced the concept of “OR-gate CARs,” which refers to a bispecific CAR that enables either antigen to independently initiate a potent T-cell response. Subsequently, the CD19‐OR‐CD20 CAR was successfully constructed, which is the first “OR‐gate CAR” capable of preventing antigen escape (18, 19). They confirmed that OR-gate CAR can effectively eradicate established tumor xenografts and prevent the downregulation of CD19 expression (19). CD19/CD20 dual-target CAR T-cells have been prepared at multiple centers and proven to be feasible (20–22). Of the various CAR T-cell therapies that are known, T-cells engineered with CAR-targeting CD19 have shown remarkable efficacy in patients with hematological malignancies, such as B-cell acute lymphoblastic leukemia (23–25) and B-cell lymphoma (26–28).

**Figure 1:**
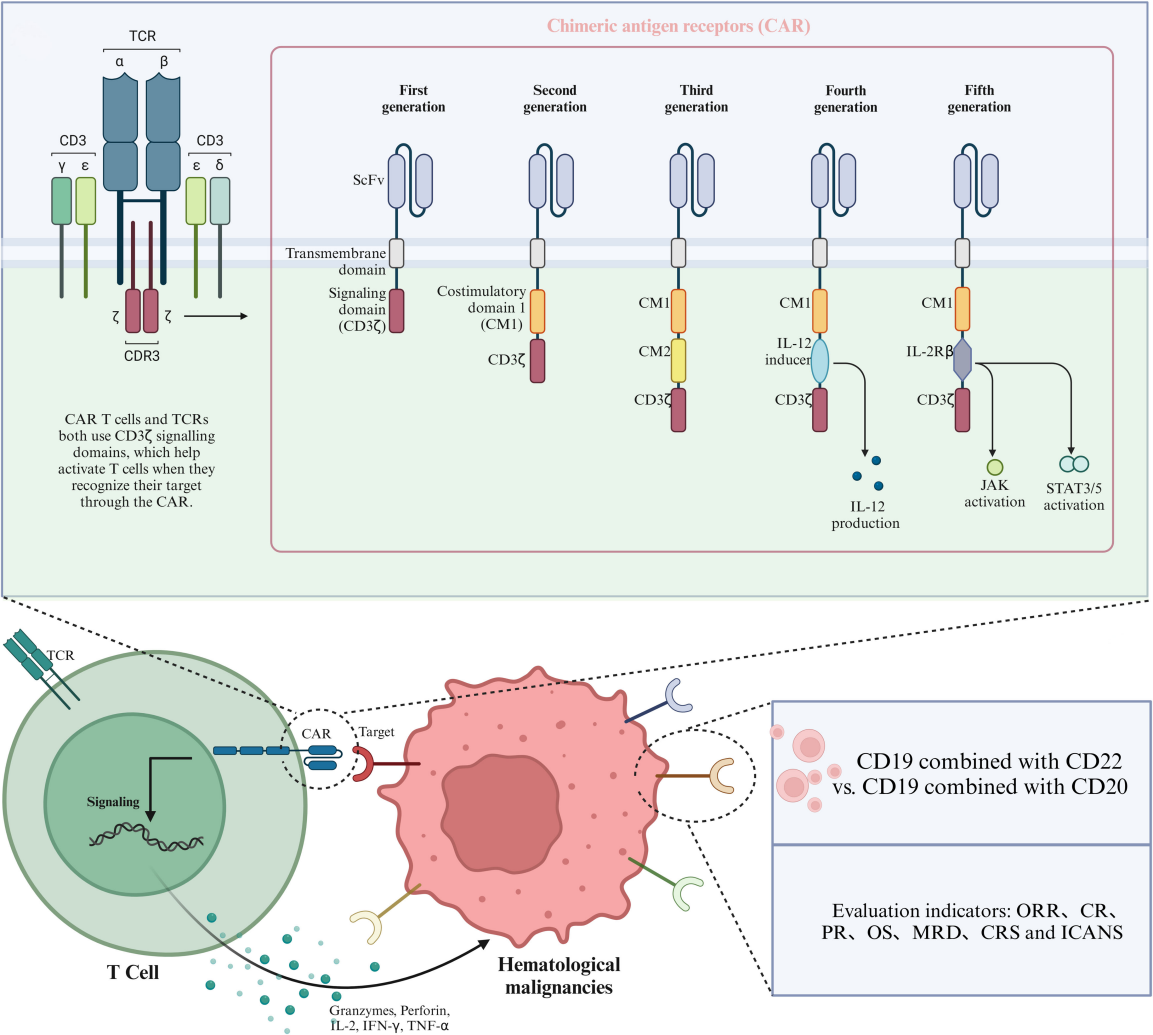
Schematic diagram of CAR structure and therapeutic targets of CAR T-cells in hematological malignancies.

This study is the latest to systematically review the efficacy of CD19 combined with CD22 or CD20 CAR T-cells for the treatment of hematological malignancies. The aim was to compare the efficacy and safety of CD19 combined with CD22 with that of CD19 combined with CD20 CAR T-cell therapy in the treatment of hematological malignancies, so as to provide an evidence-based reference for research. The flow chart of the study is shown in **Figure 1**.

## 2 Materials and methods

### 2.1 Search strategy

This study followed the Cochrane Handbook’s Preferred Reporting Project for Meta-Analysis (PRISMA) guidelines (29). The PubMed, Science Direct, Blood and medical databases and other databases were searched for all studies on the efficacy and safety of CD19 combined with CD22 or CD20 CAR T-cell therapy in patients with hematological malignancies that were published as of October 2024. The following keywords were used: CAR-T, hematological malignancies, CD19, CD22, and CD20. In addition, the reference lists of the relevant studies were thoroughly searched for relevant reviews. The literature search was conducted by two independent authors, and any disagreements that arose were resolved through discussion.

### 2.2 Inclusion and exclusion criteria

The inclusion criteria were as follows: (1) Clinical investigations on the efficacy and safety of anti-CD19 combined with CD22 or CD20 cell therapy in the treatment of hematological malignancies. (2) Reporting of the following outcomes: overall response rate (ORR), complete response (CR) rate, partial response (PR) rate, overall survival (OS) rate, minimal residual disease (MRD)-negative response rate, incidence of cytokine release syndrome (CRS), and incidence of immune effector cell-associated neurotoxicity syndrome (ICANS). We excluded (1) case reports or case series, reviews, meta-analyses, abstracts, and correspondence with unavailable data; (2) investigations on non-bispecific CAR T-cell therapy; and (3) studies with less than 9 patients.

### 2.3 Data extraction and quality assessment

A pre-designed table containing the extracted variables is presented. First author name, year of publication, number of patients, type of malignancy, type of CAR-T, efficacy outcomes (ORR, CR, PR, OS, and MRD-negative response rate), and safety outcomes (incidence of CRS and incidence of ICANS) were retrieved by two evaluators. If two or more studies covered the same group/patient subgroup, only the studies with the largest sample size or the most complete data were included to avoid duplication. The studies included in this study were all single-arm trials, and the Joanna Briggs Institute (JBI) scale was used to evaluate the quality of the included studies (30).

### 2.4 Statistical analysis

Statistical analysis was performed using the R software (version 4.4.1). We performed subgroup analyses to evaluate efficacy and safety based on the type of CAR T-cell therapy administered. *I* ^2^ was used to assess the degree of heterogeneity among the included studies. *I* ^2^ < 50% was considered to indicate acceptable heterogeneity, and the fixed-effects model was used to calculate the effectiveness rate, adverse reaction rate, and survival rate and their respective 95% confidence intervals. *I* ^2^ < 50% was considered to indicate significant heterogeneity, and the random-effects model was used to calculate the effectiveness rate, adverse reaction rate, and survival rate and their respective 95% confidence intervals. *P* < 0.05 was considered to indicate statistical significance. We performed “leave-out-one” analysis and created a forest plot to depict the sensitivity of the results. In addition, we used Egger’s test to determine funnel plot asymmetry and explore potential publication bias (31).

## 3 Results

### 3.1 Characteristics of the included studies

**Figure 2:**
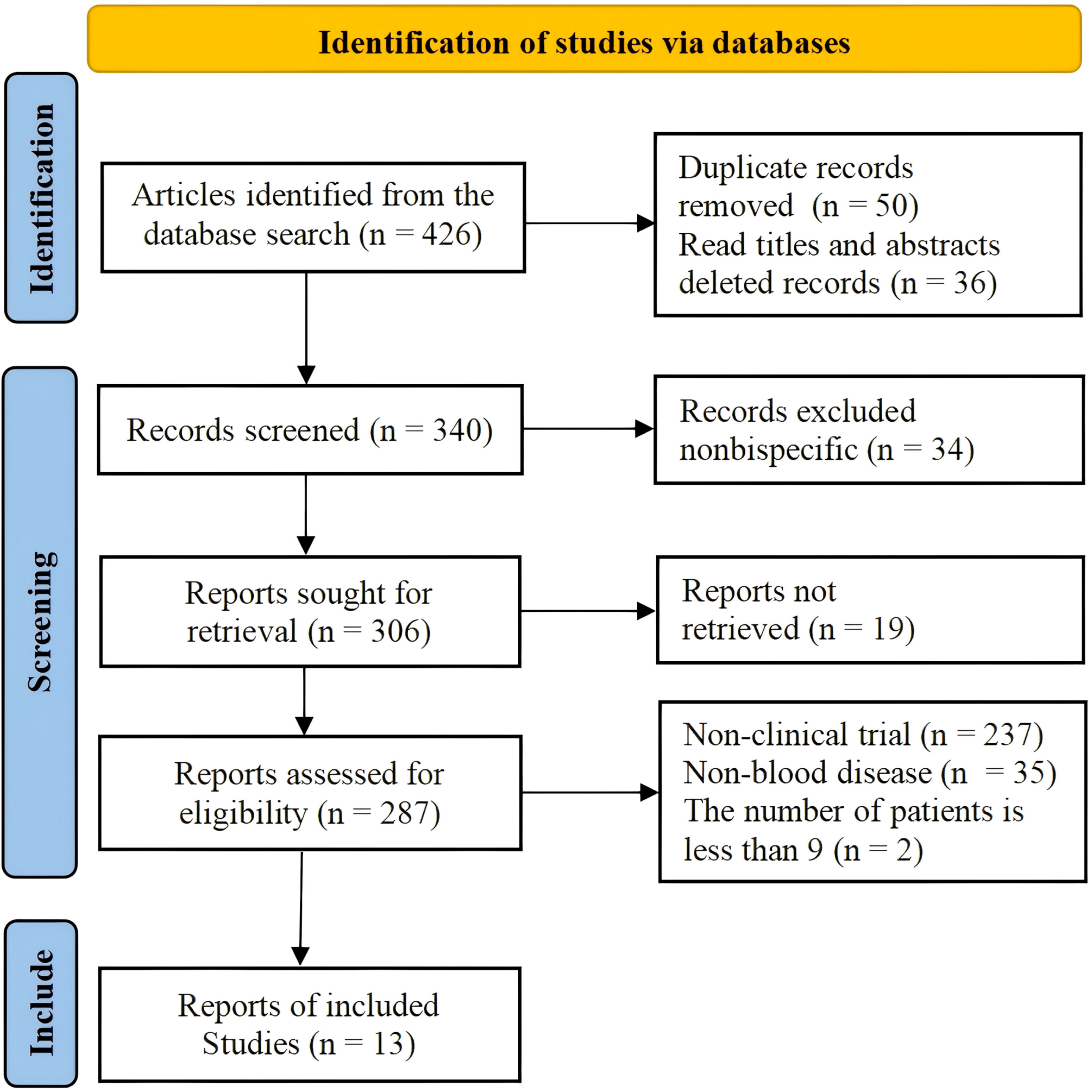
Flow chart depicting the literature search and selection process.

A total of 13 studies that met the criteria were selected, including 8 studies on the treatment of hematological malignancies with CD19 combined with CD22 CAR T-cell therapy and 5 studies on the treatment of hematological malignancies with CD19 combined with CD20 CAR T-cell therapy. The literature search and research selection process are depicted in detail in **Figure 2**, and detailed information about the characteristics of the enrolled studies is presented in **Table 1**. The JBI scale showed that all the studies were of high quality (**Supplementary Table 1**).

**Table 1:** Features included in the study.

First author	Number of patients	Type of malignancies	CAR-T type	Reported outcomes
Jay Y Spiege 202l (32)	38	B-ALL, LBCL	Both CD19 and CD22 CAR	CRS, ICANS, CR, PR, ORR, MRD
Shuangyou Liu 2021 (33)	27	R/R precursor B-ALL	Both CD19 and CD22 CAR	CRS, ICANS, CR, PR, ORR, OS, MRD
Shaun Cordoba 2021 (34)	15	R/R B-ALL	Both CD19 and CD22 CAR	CRS, ICANS, CR, ORR, OS
Haneen Shalabi 2022 (35)	20	B-ALL	Both CD19 and CD22 CAR	CRS, ICANS, CR, PR, ORR, OS, MRD
Tianyi Wang 2023 (36)	225	ALL	Both CD19 and CD22 CAR	CRS, ICANS, CR, ORR, OS, MRD
Jing Pan 2023 (37)	81	R/R B-ALL	Both CD19 and CD22 CAR	CRS, ICANS, CR, ORR, OS, MRD
Changju Qu 2022 (38)	33	R/R DLBCL	Both CD19 and CD22 CAR	CRS, ICANS, CR, PR, ORR, OS
Jiahua Niu 2023 (39)	15	MRD positive B-ALL	Both CD19 and CD22 CAR	CRS, CR, ORR, OS, MRD
Chuan Tong 2020 (40)	28	R/R NHL	Both CD19 and CD20 CAR	CRS, ICANS, CR, PR, ORR, OS
Nirav N Shah 2020 (41)	22	R B-cell malignancies	Both CD19 and CD20 CAR	CRS, ICANS, CR, PR, ORR
Wei Sang 2020 (42)	21	R/R DLBCL	Both CD19 and CD20 CAR	CRS, ICANS, CR, PR, ORR
Yajing Zhang 2022 (43)	87	R/R NHL	Both CD19 and CD20 CAR	CRS, CR, PR, ORR, OS
Joanna C Zurko 2022 (44)	16	R/R B-cell malignancies	Both CD19 and CD20 CAR	CR, PR, ORR, OS

ALL, acute lymphoblastic leukemia; B-ALL, B-cell acute lymphoblastic leukemia; LBCL, large B-cell lymphoma; Precursor B-ALL, Precursor B-cell leukemia; MRD positive B-ALL, MRD positive B-cell acute lymphoblastic leukemia; DLBCL, Diffuse large B-cell lymphoma; NHL, Non-Hodgkin’s lymphomas; ORR, Objective response rate; CR, complete response; PR, partial response; OS, Overall Survival; MRD, minimal residual disease; CRS, cytokine release syndrome; ICANS, immune effector cell-associated neurotoxicity syndrome.

### 3.2 Comparison of efficacy outcomes between CD19 combined with CD22 and CD19 combined with CD20 CAR T-cell therapy

#### 3.2.1 ORR

**Figure 3:**
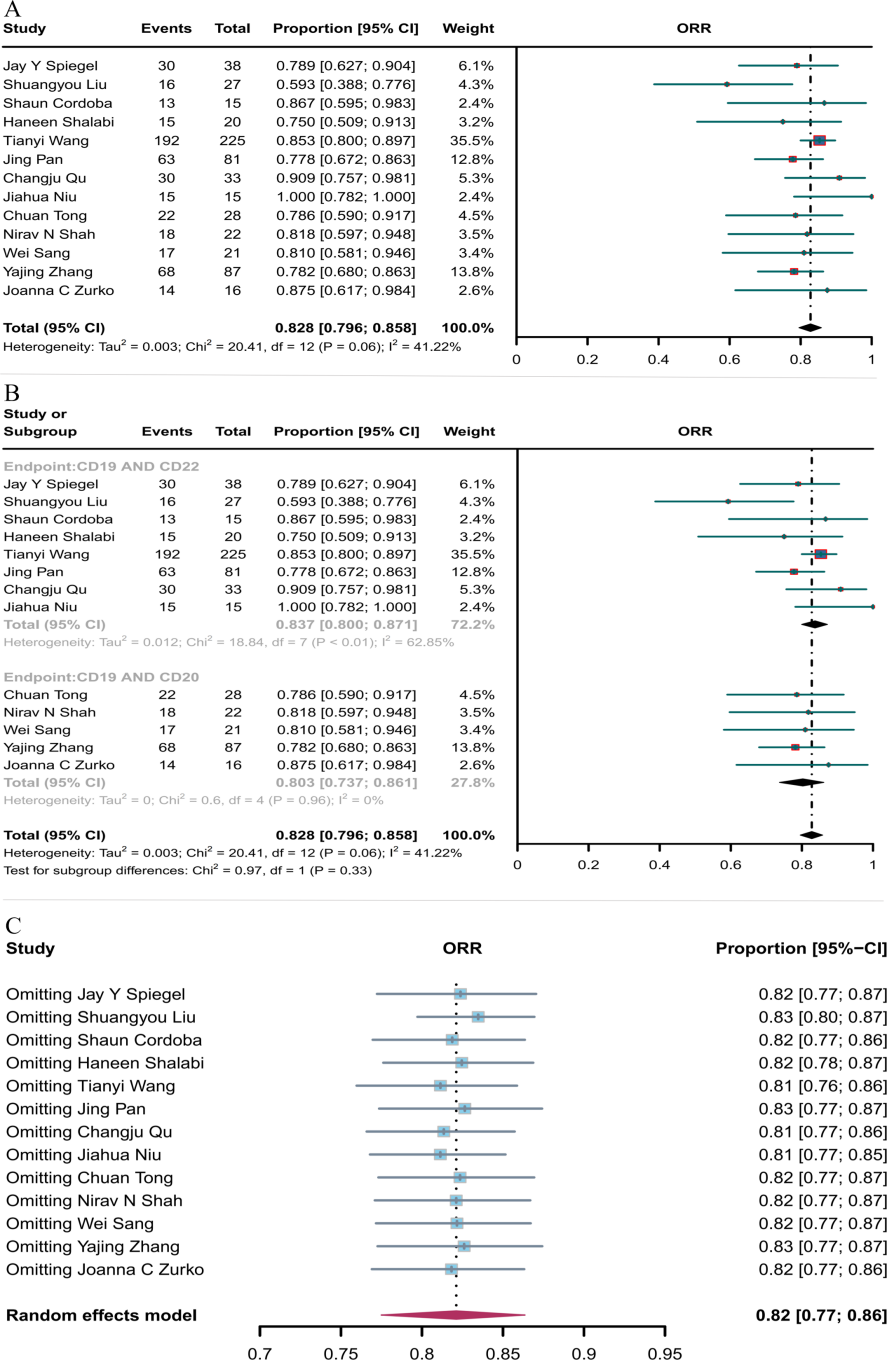
Forest map of ORR for CD19 combined with CD22 or CD20 CAR T-cell therapy. **(A)** ORR for both CD19 combined with CD22 and CD19 combined CD20 CAR T-cell therapy. **(B)** Subgroup analysis of ORR for CD19 combined with CD22 versus CD19 combined with CD20 CAR T-cells. **(C)** Forest map showing the results of sensitivity analysis with the “leave-out-one” method.

Thirteen studies reported ORR, which was 82.8% (95% CI: 79.6–85.8, **Figure 3A**) when both combination therapies were considered together. Subgroup analysis showed that the ORR of CD19 combined with CD22 CAR T-cell therapy (83.7%, 95% CI: 80.0–87.1) was better than the ORR of CD19 combined with CD20 CAR T-cell therapy (80.3%, 95% CI: 73.7–86.1, **Figure 3B**), although the difference was not significant. Sensitivity analysis showed that none of the studies significantly interfered with the results (**Figure 3C**), thus indicating the robustness of the results.

#### 3.2.2 CR

**Figure 4:**
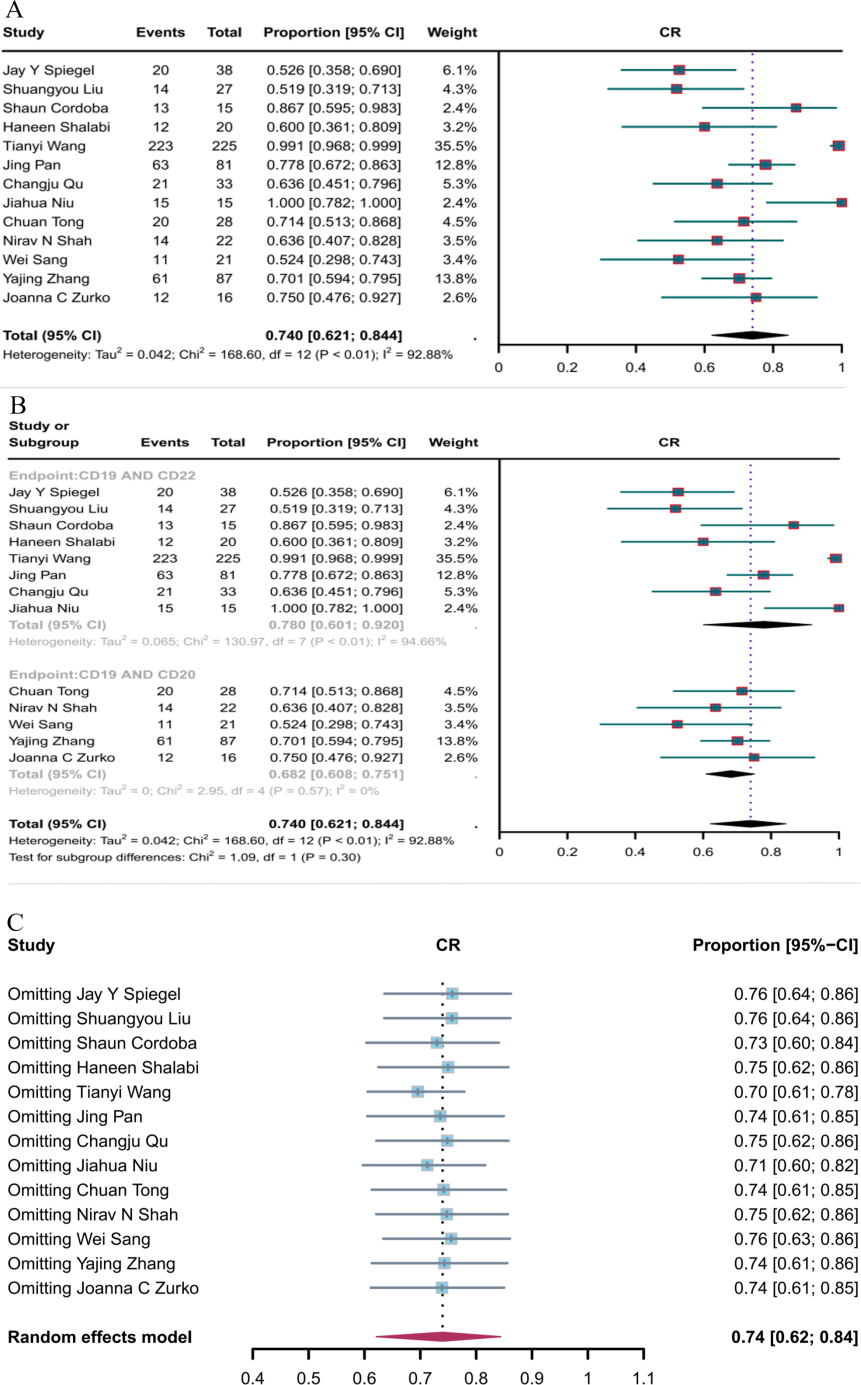
Forest map of CR for CD19 combined with CD22 or CD20 CAR T-cell therapy. **(A)** CR for both CD19 combined with CD22 and CD19 combined CD20 CAR T-cell therapy. **(B)** Subgroup analysis of CR for CD19 combined with CD22 versus CD19 combined with CD20 CAR T-cells. **(C)** Forest map showing the results of sensitivity analysis with the “leave-out-one” method.

Thirteen studies reported CR, and the overall CR rate of CD19 combined with CD22 or CD20 CAR T-cell therapy was 74.0% (95% CI: 62.1–84.4, **Figure 4A**). The CR rate of CD19 combined with CD22 CAR T-cell therapy (78.0%, 95% CI: 60.1–92.0) was (non-significantly) better than the CR rate of CD19 combined with CD20 CAR T-cell therapy (68.2%, 95% CI: 60.8–75.1, **Figure 4B**). Sensitivity analysis showed that none of the studies had a significant impact on the results, thus demonstrating the robustness of the results (**Figure 4C**).

#### 3.2.3 PR

**Figure 5:**
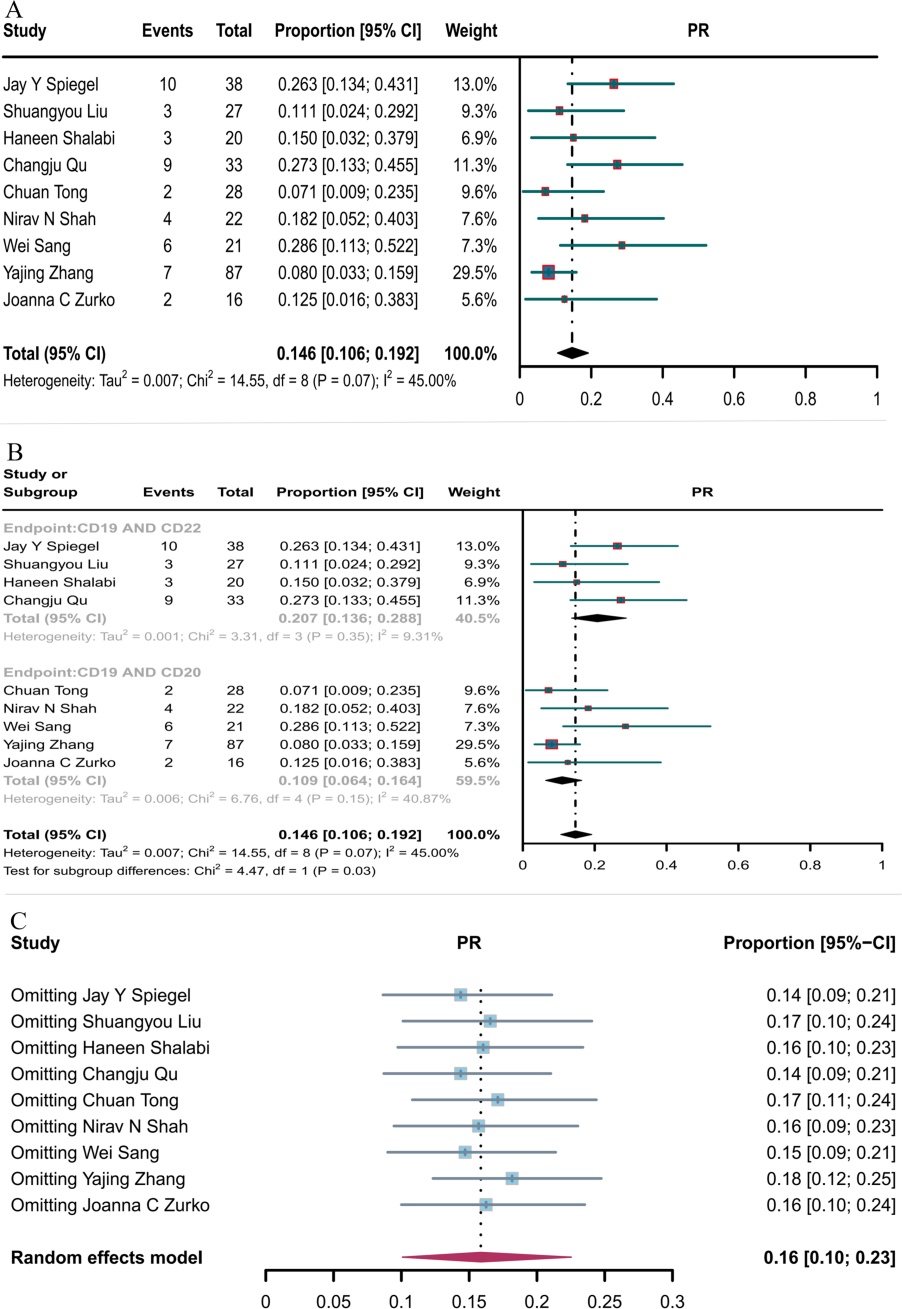
Forest map of PR for CD19 combined with CD22 or CD20 CAR T-cell therapy. **(A)** PR for both CD19 combined with CD22 and CD19 combined with CD20 CAR T-cell therapy. **(B)** Subgroup analysis of PR for CD19 combined with CD22 versus CD19 combined CD20 CAR T-cells. **(C)** Forest map showing the results of sensitivity analysis with the “leave-out-one” method.

Nine studies reported PR, with the overall PR rate of CD19 in combination with CD22 or CD20 CAR T-cell therapy being 14.6% (95% CI: 10.6–19.2, **Figure 5A**). The PR rate of CD19 combined with CD22 CAR T-cell therapy (20.7%, 95% CI: 13.6–28.8) was significantly higher (*P* = 0.03) than that of CD19 combined with CD20 CAR T-cell therapy (10.9%, 95% CI: 6.4–16.4, **Figure 5B**). Subsequent sensitivity analysis observed that no single study had a significant impact on the results (**Figure 5C**), thus demonstrating the stability of the current results.

#### 3.2.4 OS

**Figure 6:**
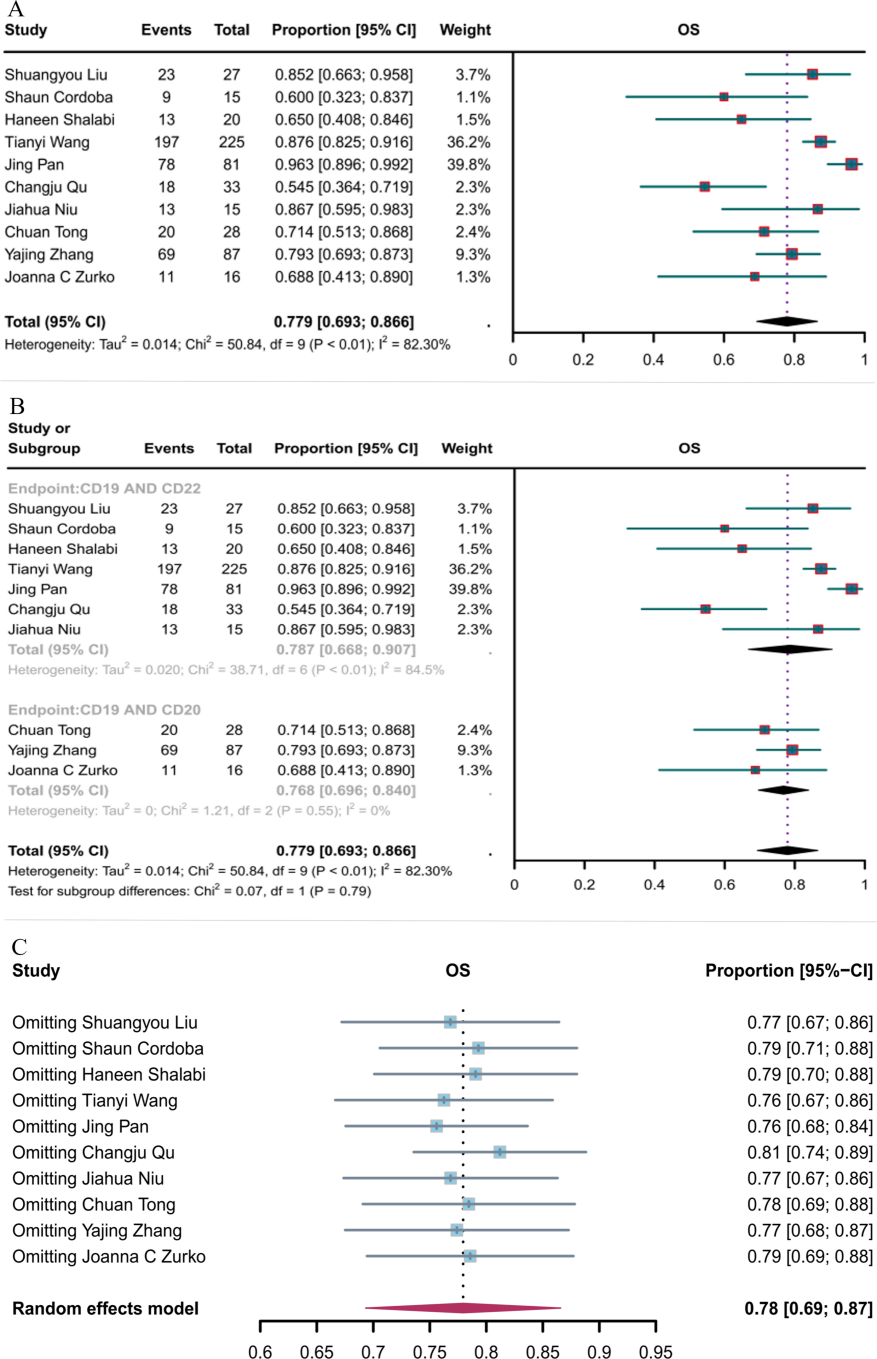
Forest map of OS for CD19 combined with CD22 or CD20 CAR T-cell therapy. **(A)** OS for both CD19 combined with CD22 and CD19 combined with CD20 CAR T-cell therapy. **(B)** Subgroup analysis of OS for CD19 combined with CD22 versus CD19 combined with CD20 CAR T-cells. **(C)** Forest map showing the results of sensitivity analysis with the “leave-out-one” method.

Ten studies reported OS. The overall OS rate for hematological malignancies treated with CD19 combined with CD22 or CD20 CAR T-cell therapy was 77.9% (95% CI: 69.3–86.6, **Figure 6A**). The OS rate for hematological malignancies treated with CD19 combined with CD22 CAR T-cell therapy (78.7%, 95% CI: 66.8–90.7) was greater, although not significantly, than that for hematological malignancies treated with CD19 combined with CD20 CAR T-cell therapy (76.8%, 95% CI: 69.6–84.0, **Figure 6B**). Sensitivity analysis observed that no single study had a significant impact on the results (**Figure 6C**); thus, the current results can be considered as robust.

#### 3.2.5 MRD-negative response rate

**Figure 7:**
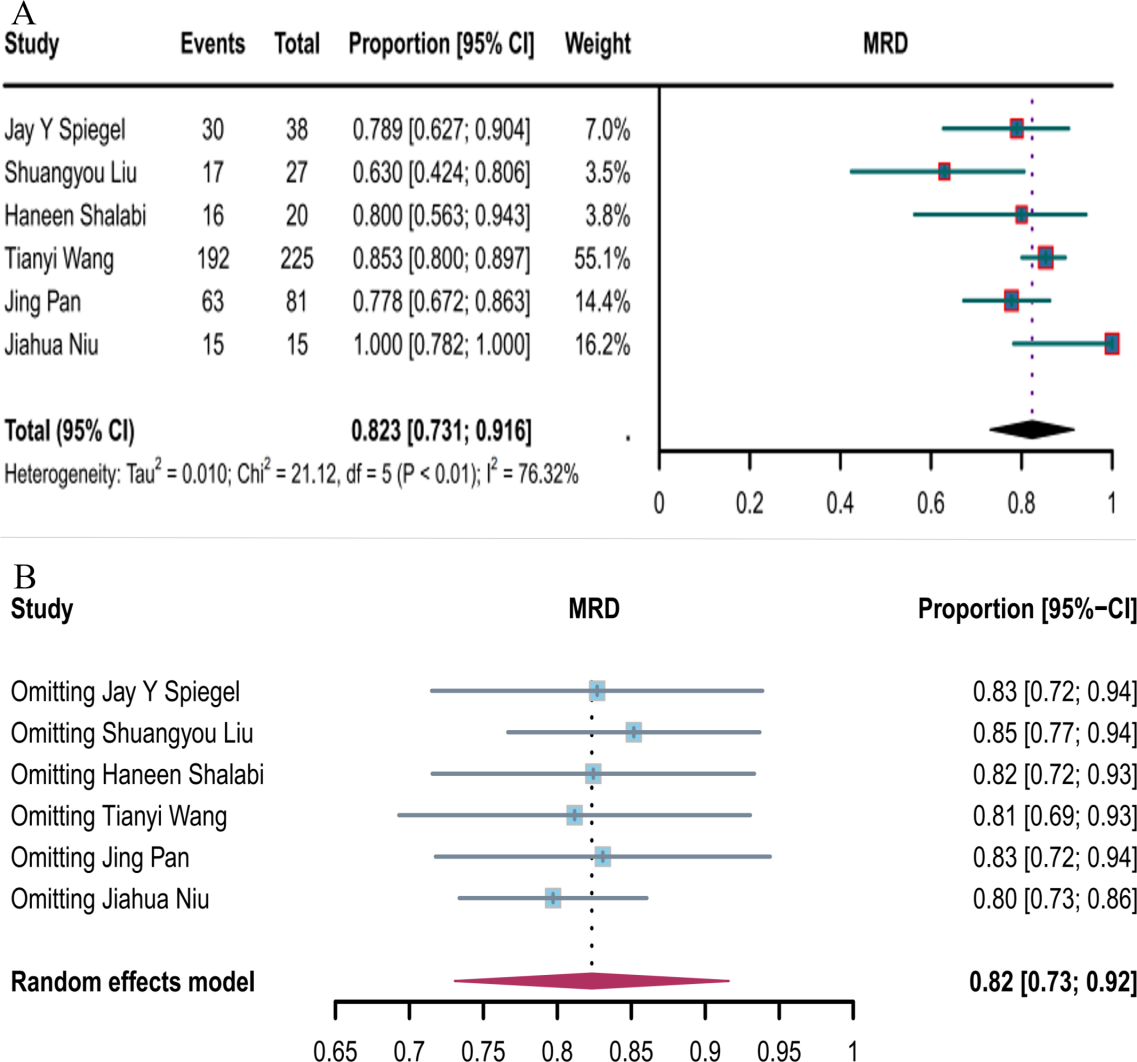
Forest map of MRD-negative response to CD19 combined with CD22 CAR T-cell therapy. **(A)** MRD-negative response rate for CD19 combined with CD22 CAR T-cell therapy. **(B)** Forest map showing the results of sensitivity analysis with the “leave-out-one” method.

Six studies reported the negative MRD response rates for CD19 combined with CD22 CAR T-cells (82.3%, 95% CI: 73.1–91.6, **Figure 7A**). Sensitivity analysis showed that none of the studies significantly interfered with the results (**Figure 7B**), thus implying the stability of the current results.

### 3.3 Comparison of safety outcomes between CD19 combined with CD22 and CD19 combined with CD20 CAR T-cell therapy

#### 3.3.1 Incidence of CRS

**Figure 8:**
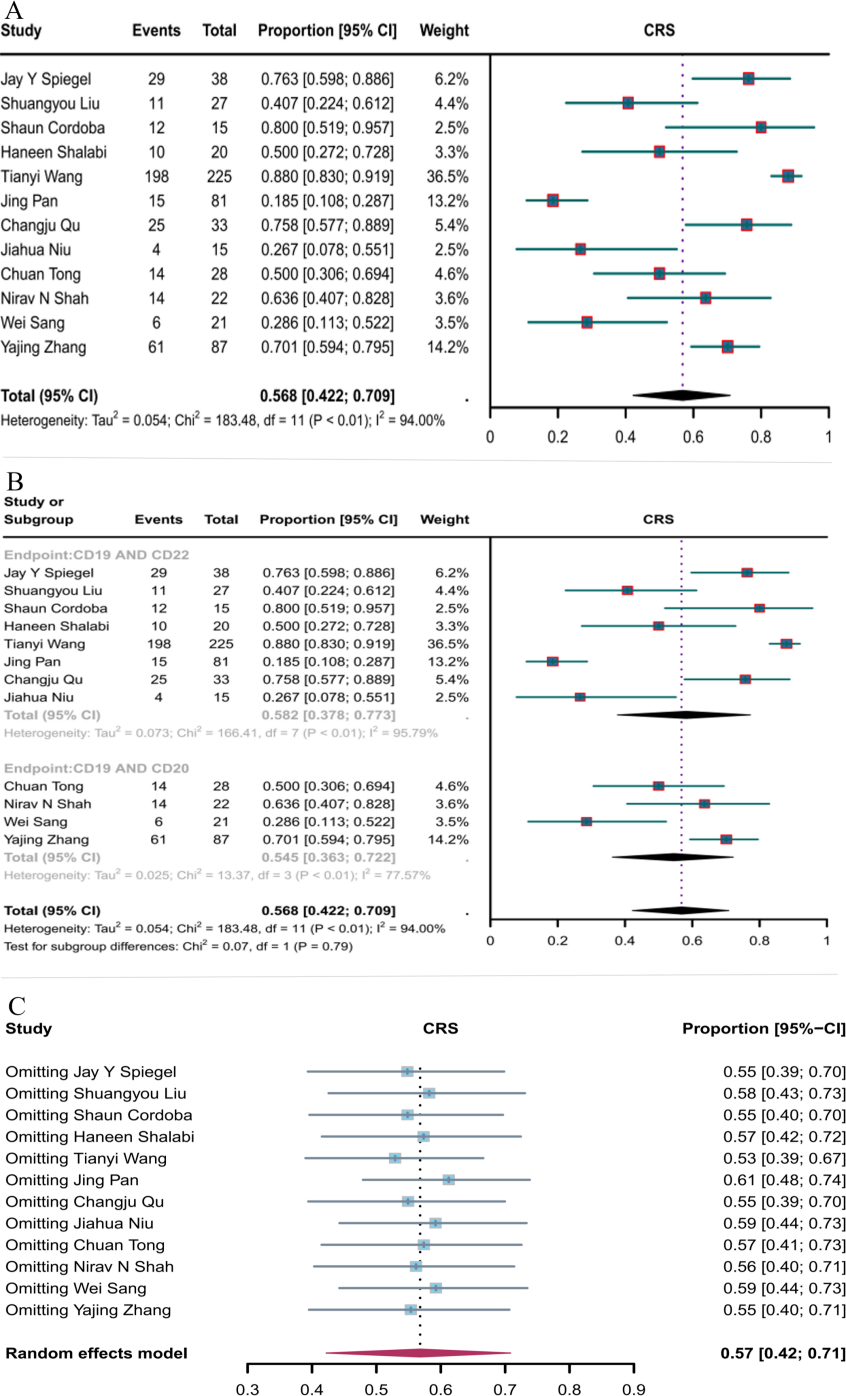
Forest map of the incidence of CRS for CD19 combined with CD22 or CD20 CAR T-cell therapy. **(A)** Incidence of CRS for both CD19 combined with CD22 and CD19 combined with CD20 CAR T-cell therapy. **(B)** Subgroup analysis of the incidence of CRS for CD19 combined with CD22 versus CD19 combined with CD20 CAR T-cells. **(C)** Forest map showing the results of sensitivity analysis with the “leave-out-one” method.

Twelve studies reported the incidence of CRS. The overall CRS rate of CD19 combined with CD22 or CD20 CAR T-cell therapy for hematological malignancies was 56.8% (95% CI: 42.2–70.9, **Figure 8A**). The incidence of CRS with CD19 combined with CD22 CAR T-cell therapy (58.2%, 95% CI: 37.8–77.3) was higher than that with CD19 combined with CD20 CAR T-cell therapy (54.5%, 95% CI: 36.3–72.2, **Figure 8B**), although the difference was not significant. Sensitivity analysis showed that none of the studies significantly interfered with the results (**Figure 8C**). Thus, the current results are stable.

#### 3.3.2 Incidence of ICANS

**Figure 9:**
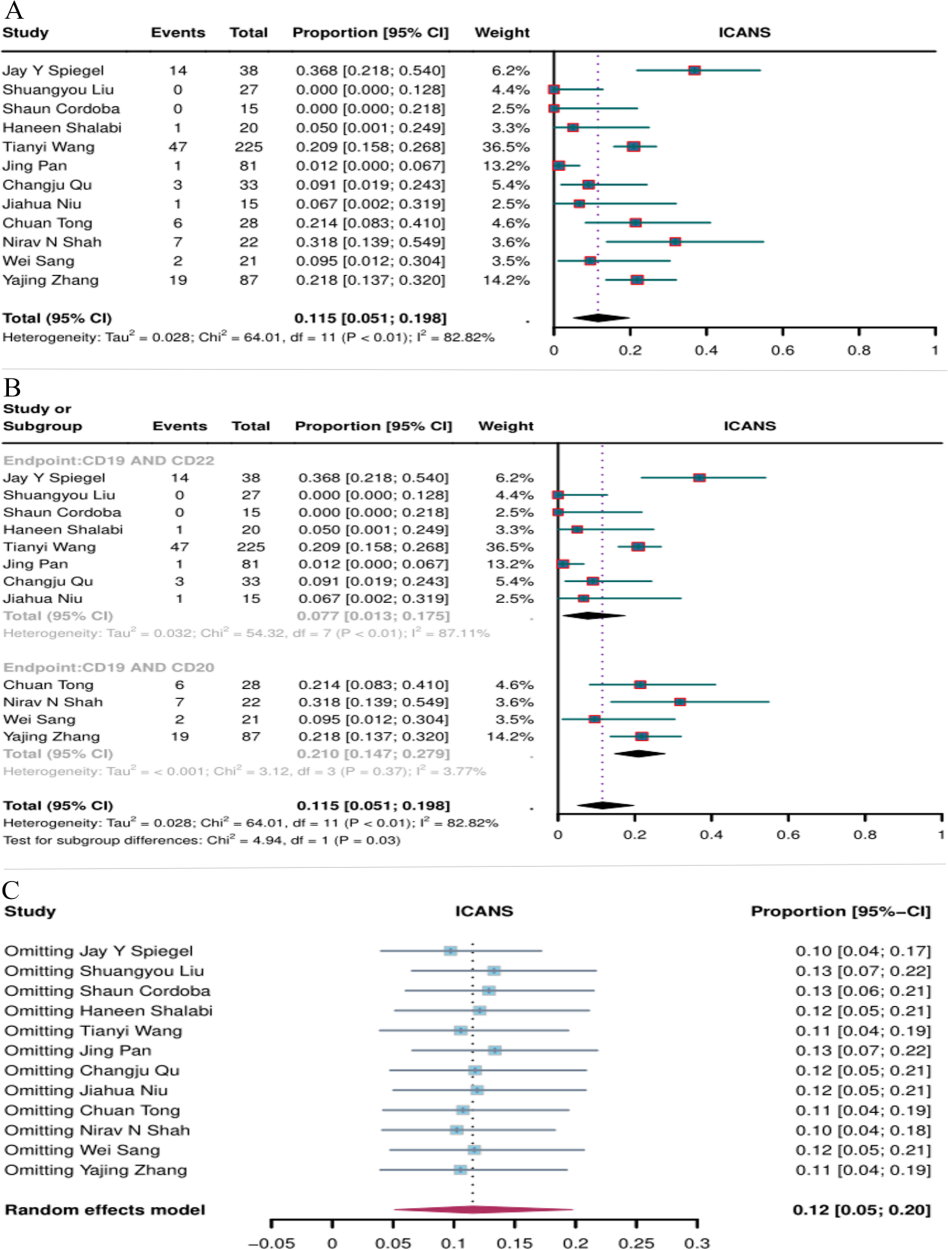
Forest map of the incidence of ICANS for CD19 combined with CD22 or CD20 CAR T-cell therapy. **(A)** Incidence of ICANS for both CD19 combined with CD22 and CD19 combined with CD20 CAR T-cell therapy. **(B)** Subgroup analysis of the incidence of ICANS for CD19 combined with CD22 versus CD19 combined with CD20 CAR T-cells. **(C)** Forest map showing the results of sensitivity analysis with the “leave-out-one” method.

The incidence of ICANS was reported in 12 studies. The overall ICANS rate of CD19 combined with CD20 or CD22 CAR T-cell therapy for hematological malignancies was 11.5% (95% CI: 5.1–19.8, **Figure 9A**). The incidence of ICANS was significantly lower with CD19 combined with CD22 CAR T-cell therapy (7.7%, 95% CI: 1.3–17.5) than with CD19 combined with CD20 CAR T-cell therapy (21%, 95% CI: 14.7–27.9, **Figure 9B**) (*P* = 0.03). Sensitivity analysis showed that none of the studies significantly interfered with the results (**Figure 8C**), demonstrating the stability of the current results.

### 3.4 Publication bias

The risk of publication bias was assessed through funnel plot analysis and Egger’s tests (**Supplementary Figure 1**, **Supplementary Table 2**). No evidence of potential publication bias was observed for PR, OS, or CRS at the last follow-up based on visual inspection of the plot and Egger’s tests. However, potential publication bias was identified for ORR, CR, MRD-negative response rate, and incidence of ICANS.

## 4 Discussion

This study synthesized the current evidence on combined therapy with CD19 and CD20 or CD22 CAR T-cells and compared the efficacy and safety of combining CD19 with CD20 or CD22 CAR T-cells for the treatment of hematological malignancies. A total of 13 clinical trials were included in this study. According to subgroup analysis, the PR of CD19 combined with CD20 in the treatment of hematological malignancies was lower than those of CD19 combined with CD22. The possible reason for this is that some B-cell malignancies can evade the attack of the immune system by down-regulating CD20 (45), leading to the failure of CD20 CAR T-cell therapy. In contrast, the down regulation of CD22 is less common (46). Although CD20 expression is low or down-regulated in B-cell malignancies, CD20 expression is high and stable in other subtypes, such as follicular lymphoma, and CD20 CAR T-cell therapy may still be effective in these patients (47). In addition, local regions or subclones may retain CD20 positivity even when overall CD20 expression is low, and evaluating CD20 CAR-T response can identify these “residual sensitive clones” and provide a basis for combination therapy (48). By analyzing low-response cases, CD20 CAR affinity (to avoid over-activation leading to T-cell depletion) or co-stimulatory domain design can be optimized to increase efficacy and provide help for clinical treatment (49).

CRS and ICANS are adverse effects linked to CAR T-cell therapy, which significantly restrict its widespread application. CRS, the most significant CAR-T toxicity, is an inflammatory syndrome induced by a multitude of cytokines generated by both CAR T-cells themselves and other cells. It is characterized by low blood pressure, fever, and tachycardia, among many other abnormalities (50). ICANS is also known as a neurotoxic complication and is the second most frequent adverse event of CAR T-cell therapy, it can occur simultaneously with or after presentation of CRS (51). Subgroup analysis in this study showed that the incidence of ICANS was significantly lower with CD19 combined with CD22 CAR T-cell therapy compared to CD19 combined with CD20. This may occur because CD19 combined with CD22 has more efficient tumor clearance and more precise T-cell functional activation. Researches show that CD22 is usually expressed at a significantly higher density in malignant B-cells than in normal B-cells (especially in relapsed/refractory patients), whereas CD20 is widely distributed in both normal and malignant B-cells (52). CD19/CD22 dual-target CAR T is more likely to target tumor cells with high CD22 expression, reduce overkill of normal B-cells, and thus reduce the total amount of systemic inflammatory factors (such as IL-6, IFN-γ) released (53). In addition, most CD22 CAR-T uses the 4-1BB costimulatory domain, while some CD20 CAR-T uses the CD28 domain. The 4-1BB signal is more likely to promote memory T-cell formation and persistence, while the CD28 domain drives stronger short-term activation, possibly leading to more intense initial cytokine release (54). Many studies have shown that the toxicity of CAR T-cell therapy is caused by multiple factors, including early and peak levels of certain cytokines, peak blood CAR T-cell levels, CAR T-cell dose, CAR design, and patient disease burden (55–57). In the future, further understanding of these toxic mechanisms will be important to reduce the toxicity of bisspecific CAR T-cells (58).

In summary, our findings indicate that the combination of CD19 with either CD22 or CD20 CAR T-cell therapy yields a substantial and enduring clinical response in patients afflicted with hematological malignancies. There are certain limitations of this study that need to be acknowledged. We observed heterogeneity in CR, OS, MRD, CRS, and ICANS indicators. We reduced the impact of heterogeneity by using a random-effects model and assessed the quality of the literature using the JBI scale (30). Another limitation was that we did not analyze progression-free survival because most of the included clinical trials are ongoing. Future clinical trials with larger sample sizes and extended follow-up periods will help provide a clearer understanding of the efficacies of combined and bispecific CAR T-cell therapies.

## 5 Conclusion

This study explored the synergistic anti-tumor effects of CD19 combined with CD22 or CD20 dual-target CAR T-cell therapy in hematologic malignancies, and its preliminary results showed that CD19 combined with CD22 CAR T-cell therapy had a higher partial response rate in the treatment of hematologic malignancies than CD19 combined with CD20. In addition, CD19 and CD22 CAR T-cell therapy has a higher safety profile in the occurrence of ICANS than the combination of CD19 and CD20. It is important to note that dual-targeted CAR-T therapy has not yet received regulatory approval, and its long-term safety, population applicability, and sequential integration with existing therapies still need to be validated by larger clinical trials. However, these data still provide an important clinical basis for the development of new therapeutic targets and the construction of therapeutic methods for the treatment of hematologic malignancies, and broaden our understanding of CD19 dual-targeted CAR T therapy.

## Data Availability

The original contributions presented in the study are included in the article/**Supplementary Material**. Further inquiries can be directed to the corresponding authors.

## Supplementary material

Supplementary Figure 1 Funnel plots depicting publication bias in the reported outcomes of CD19 combined with CD22 or CD20 CAR T-cell therapy. **(A)** ORR, **(B)** CR, **(C)** PR, **(D)** OS, **(E)** MRD-negative response, **(F)** CRS, and **(G)** ICANS.
