# Supplementary figures and images for: Efficacy and safety of CD19 combined with CD22 or CD20 chimeric antigen receptor T-cell therapy for hematological malignancies
Source: Front Immunol. 2025 May 13;16:1577360. doi: 10.3389/fimmu.2025.1577360 (PMC12106392; doi:10.3389/fimmu.2025.1577360)

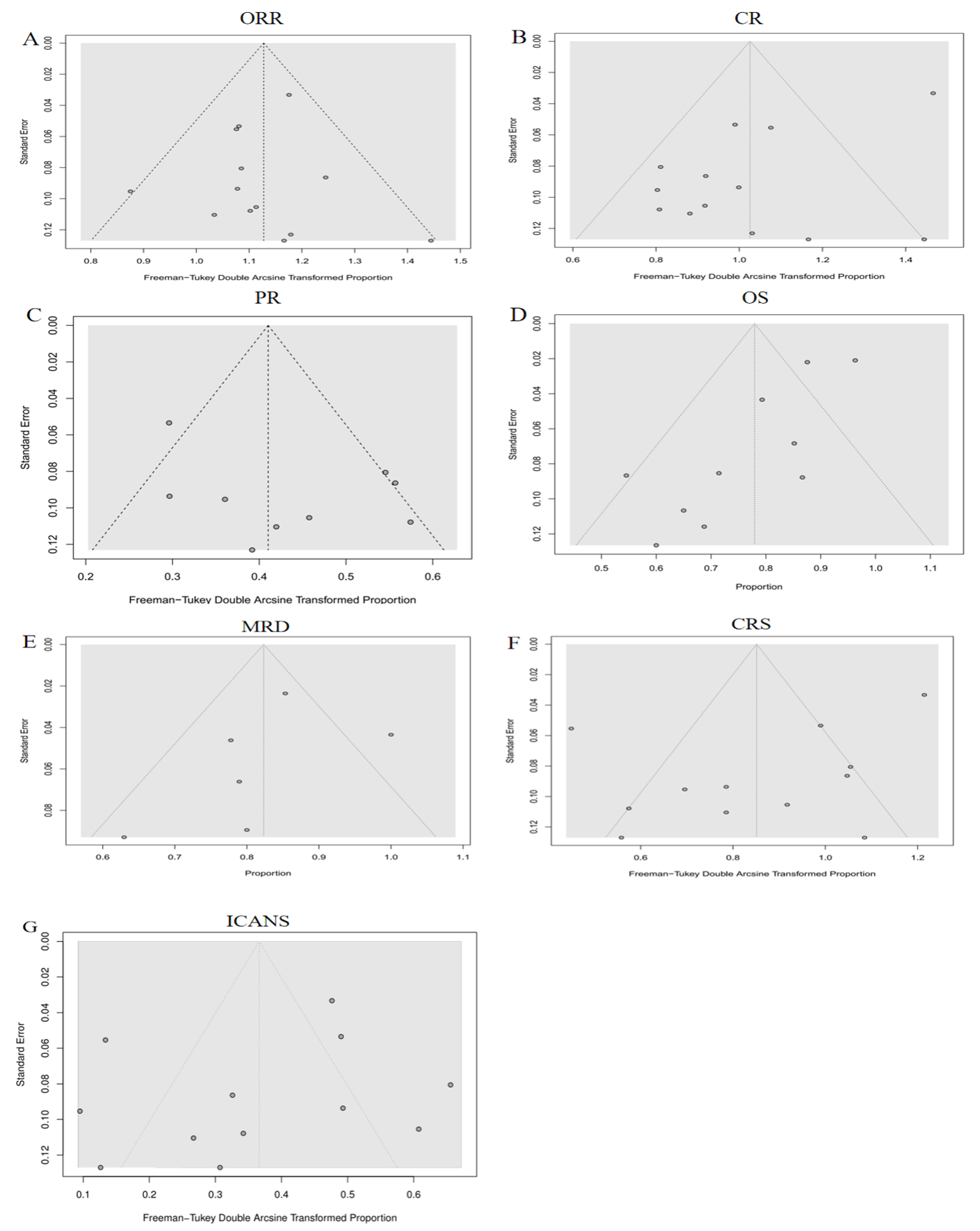

Supplement: Supplementary Figure 1 — Funnel plots depicting publication bias in the reported outcomes of CD19 combined with CD22 or CD20 CAR T-cell therapy. (A) ORR, (B) CR, (C) PR, (D) OS, (E) MRD-negative response, (F) CRS, and (G) ICANS. [file Image1.tif]
