# Supplementary material for: Efficacy and safety of CD19 combined with CD22 or CD20 chimeric antigen receptor T-cell therapy for hematological malignancies
Source: Front Immunol. 2025 May 13;16:1577360. doi: 10.3389/fimmu.2025.1577360 (PMC12106392; doi:10.3389/fimmu.2025.1577360)
Supplement: Supplementary file 2 [file Table1.docx]

Supplementary Table1. Quality assessment of JBI scale.

| Study | 1 | 2 | 3 | 4 | 5 | 6 | 7 | 8 | 9 | 10 |
| --- | --- | --- | --- | --- | --- | --- | --- | --- | --- | --- |
| Jay Y Spiegel 2021 [32] | N | Y | N | Y | N | Y | Y | Y | N | Y |
| Shuangyou Liu 2021 [33] | Y | Y | N | Y | N | Y | Y | Y | N | Y |
| Shaun Cordoba 2021 [34] | Y | N | Y | Y | Y | Y | Y | Y | N | Y |
| Haneen Shalab 2022 [35] | N | Y | N | Y | N | Y | Y | Y | N | Y |
| Tianyi Wang 2023 [36] | N | N | N | Y | Y | Y | N | Y | N | Y |
| Jing Pan 2023 [37] | Y | Y | N | Y | N | Y | Y | Y | Y | Y |
| Changju Qu 2022 [38] | N | Y | N | Y | N | Y | Y | Y | N | Y |
| Jiahua Niu 2023 [39] | N | Y | N | Y | N | Y | Y | Y | N | N |
| Chuan Tong 2020 [40] | N | Y | N | Y | N | Y | Y | Y | N | N |
| Nirav N Shah 2020 [41] | N | Y | N | Y | N | Y | Y | Y | N | N |
| Wei Sang 2020 [42] | Y | Y | N | Y | N | Y | Y | Y | N | Y |
| Yajing Zhang 2022 [43] | Y | Y | N | Y | N | Y | Y | Y | N | Y |
| Joanna C Zurko 2022 [44] | N | N | N | Y | Y | Y | Y | Y | N | N |

Note: (1) Were there clear criteria for inclusion in the case series?; (2) Was the condition measured in a standard, reliable way for all participants included in the case series?; (3) Were valid methods used for identification of the condition for all participants included in the case series?; (4) Did the case series have consecutive inclusion of participants?; (5) Did the case series have complete inclusion of participants?; (6) Was there clear reporting of the demographics of the participants in the study?; (7) Was there clear reporting of clinical information of the participants?; (8) Were the outcomes or follow up results of cases clearly reported?; (9) Was there clear reporting of the presenting site (s) /clinic (s) demographic information?; (10) Was statistical analysis appropriate?.

Response Key: Y: Yes; N: No.
