# Supplementary material for: Efficacy and safety of CD19 combined with CD22 or CD20 chimeric antigen receptor T-cell therapy for hematological malignancies
Source: Front Immunol. 2025 May 13;16:1577360. doi: 10.3389/fimmu.2025.1577360 (PMC12106392; doi:10.3389/fimmu.2025.1577360)
Supplement: Supplementary file 3 [file Table2.docx]

Supplementary Table 2. The pooled proportions of outcomes for hematological malignancies with CD19 in combination with CD22 or CD20 CAR-T cell therapy

| Outcomes | No. of studies | Patients  (n/N) | Pooled proportion (%) | 95% CI | Heterogeneity within study | | Publication bias |
| --- | --- | --- | --- | --- | --- | --- | --- |
|  |  |  |  |  | I^2^(%) | p-value | p-value |
| ORR | 13 | 513/628 | 82.8% | [0.796; 0.858] | 41.22% | =0.06 | < 0.05 |
| CR | 13 | 499/628 | 74% | [0.621; 0.844] | 92.88% | < 0.01 | < 0.05 |
| PR | 9 | 46/292 | 14.6% | [0.106; 0.192] | 45% | =0.07 | >0.05 |
| OS | 10 | 451/547 | 77.9% | [0.693; 0.866] | 82.3% | < 0.01 | >0.05 |
| MRD | 6 | 333/406 | 82.3% | [0.731; 0.916] | 76.32% | < 0.01 | <0.05 |
| CRS | 12 | 399/612 | 56.8% | [0.422; 0.709] | 94% | < 0.01 | >0.05 |
| ICANS | 12 | 101/612 | 11.5% | [0.051; 0.198] | 82.82% | < 0.01 | <0.05 |

Abbreviations: CAR-T cell: chimeric antigen receptor T cell; ORR: Objective response rate; CR: complete response; PR: partial response; OS: Overall Survival; MRD, minimal residual disease; CRS: cytokine release syndrome; ICANS: immune effector cell-associated neurotoxicity syndrome.
